# Saroglitazar suppresses the hepatocellular carcinoma induced by intraperitoneal injection of diethylnitrosamine in C57BL/6 mice fed on choline deficient, l-amino acid- defined, high-fat diet

**DOI:** 10.1186/s12885-023-10530-0

**Published:** 2023-01-17

**Authors:** Suresh R. Giri, Bibhuti Bhoi, Chitrang Trivedi, Akshyaya Rath, Rohan Rathod, Anish Sharma, Ramchandra Ranvir, Shekhar Kadam, Kailash Ingale, Hiren Patel, Abraham Nyska, Mukul R. Jain

**Affiliations:** 1 Department of Pharmacology and Toxicology, Zydus Research Centre, Zydus Lifesciences Limited (formerly known as Cadila Healthcare Limited), Sarkhej-Bavla N.H.No. 8A, Moraiya, Ahmedabad, Gujarat 382213 India; 2 Tel Aviv University, Yehuda HaMaccabi 31, floor 5, 6200515 Tel Aviv, Israel

**Keywords:** Hepatocellular carcinoma, NAFLD, NASH

## Abstract

**Background:**

Saroglitazar is a novel PPAR-α/γ agonist with predominant PPAR-α activity. In various preclinical models, saroglitazar has been shown to prevent & reverse symptoms of NASH. In view of these observations, and the fact that NASH is a progressive disease leading to HCC, we hypothesized that saroglitazar may prevent the development of HCC in rodents.

**Methods:**

HCC was induced in C57BL/6 mice by a single intraperitoneal injection of 25 mg/kg diethylnitrosamine (DEN) at the age of 4 weeks and then feeding the animal a choline-deficient, L-amino acid- defined, high-fat diet (CDAHFD) for the entire study duration. Eight weeks after initiation of CDAHFD, saroglitazar (1 and 3 mg/kg) treatment was started and continued for another 27 weeks.

**Results:**

Saroglitazar treatment significantly reduced the liver injury markers (serum ALT and AST), reversed hepatic steatosis and decreased the levels of pro-inflammatory cytokines like TNF-α in liver. It also resulted in a marked increase in serum adiponectin and osteopontin levels. All disease control animals showed hepatic tumors, which was absent in saroglitazar (3 mg/kg)- treatment group indicating 100% prevention of hepatic tumorigenesis. This is the first study demonstrating a potent PPARα agonist causing suppression of liver tumors in rodents, perhaps due to a strong anti-NASH activity of Saroglitazar that overrides its rodent-specific peroxisome proliferation activity.

**Conclusion:**

The data reveals potential of saroglitazar for chemoprevention of hepatocellular carcinoma in patients with NAFLD/NASH.

**Supplementary Information:**

The online version contains supplementary material available at 10.1186/s12885-023-10530-0.

## Background

Hepatocellular carcinoma (HCC) is the fifth most common tumor worldwide and the second most common cause of cancer-related death. Nonalcoholic fatty liver disease (NAFLD) and the metabolic syndrome contribute numerically more to the burden of HCC than any other risk factor including HCV infection, which is primarily due to the high prevalence of NAFLD in the population worldwide. Although a number of therapeutic options have been explored for the management of NAFLD/NASH, no pharmacological treatment is yet approved by USFDA. NAFLD is a liver disease associated with obesity, insulin resistance, type 2 diabetes mellitus (T2DM) and metabolic syndrome and its prevalence is proportional to the increase in body mass index BMI [1]. In the general population, NAFLD prevalence is 25% and it increases to 90% in obese individuals and 60% in T2DM patients [1]. NAFLD is a progressive liver disease triggered by excessive accumulation of triglycerides in hepatocyte leading to hepatic steatosis [nonalcoholic fatty liver (NAFL)], to lipotoxicity plus inflammation and hepatocyte injury [nonalcoholic steatohepatitis (NASH)] and finally to hepatic fibrosis and cirrhosis and/or HCC [2, 3].

Peroxisome proliferator-activated receptors (PPARs) are nuclear receptors that play key roles in the regulation of metabolic homeostasis, inflammation, cellular growth and differentiation [4]. Saroglitazar is a novel PPAR-α/γ dual agonist has shown to reduce lipid levels (triglycerides and cholesterol), improve insulin sensitivity, reduce liver injury, inflammation and oxidative stress which ultimately reverse the fibrosis and overall improvement in NAFLD/NASH [5–8]. It has been reported that saroglitazar significantly decreased ALT and improved steatosis, insulin resistance, and dyslipidemia in patients with NAFLD [9, 10]. Saroglitazar is approved in India for Non-Cirrhotic Non-Alcoholic SteatoHepatitis (NASH) and NAFLD and it has become first approved drug anywhere in the world for treatment of NASH and NAFLD. PPAR agonists are known to cause rodent specific HCC [11–13], however in the view of improvement of NASH symptoms by saroglitazar in rodent models and clinical trials it was hypothesized that saroglitazar, PPAR-α/γ dual agonist may prevent development of NASH mediated HCC.

## Materials and methods

### Chemicals

Saroglitazar [(S)-α-ethoxy-4-{2-[2-methyl-5-(4-methylthio) phenyl)] − 1H-pyrrol− 1-yl]- ethoxy}) benzenepropanoic acid magnesium salt] (supplementatry information Fig. F1), was supplied by Zydus Lifesciences Limited (formerly known as Cadila Healthcare Limited), Ahmedabad, India.

### Animal experiment

Four-week-old male C57BL/6 mice bred at the Zydus Research Centre (ZRC) were used in this study. The total number of mice used in this study was 102 with body weight 12.5 to 19.0 g. The animals were housed in individually ventilated cages and maintained on a standard laboratory rodent diet (Teklad 2018C, Harlan Laboratories, USA) and water ad libitum in a temperature (25 ± 3 °C) and humidity (50–70%)-controlled environment with a 12-h/12-h dark-light cycle. The experimental facility was accredited by AAALAC International (Association for Assessment and Accreditation of Laboratory Animal Care International) and all animal experiment protocols were approved by Institutional Animal Ethics Committee (IAEC) of Zydus Lifesciences Limited, Zydus Research Centre under protocol no. ZRC/PH/BP/039/10-2 K17. After acclimatization for 3 days, 92 animals were injected with DEN (25 mg/kg) and 10 animals were injected with saline by intraperitoneal route. During experimental phase, DEN administered animals were maintained on a choline deficient, L-amino acid-defined, high-fat diet (CDAHFD) containing 45 kcal% fat with 0.1% methionine (Product # A06071309, Research Diet, New Brunswick, NJ, USA). The saline administered animals were maintained on the control for CDAHF diet containing 10 kcal% fat and crystalline amino acids (Product # A06071322; Research Diet, New Brunswick, NJ, USA); saline-control diet + Vehicle (Normal control). Eight weeks of CDAHFD feeding was reported to cause liver steatosis, inflammation and stage 1 fibrosis [7]. Therefore, after eight weeks of diet all the animals were bled under isoflurane anesthesia to estimate serum biochemical parameters for liver function ALT and AST levels (day-0 or pretreatment analysis). Animals with increased serum ALT level were selected and randomized into different treatment groups (*n* = 10–15) based on serum ALT levels and body weights in such a way that mean ALT value and body weight were not significantly different between the groups. DEN with CDAHFD treated animals were divided into three groups (*n* = 15); DEN-CDAHFD + Vehicle (Disease control), DEN-CDAHFD + Saroglitazar 1 mg/kg,p.o. (D-Saro 1 mg/kg,p.o.) and DEN-CDAHFD + Saroglitazar 3 mg/kg,p.o. (D-Saro 3 mg/kg,p.o.). From next day (Day-1) onwards animals were administered with either vehicle, saroglitazar 1 mg/kg or saroglitazar 3 mg/kg, once daily orally for 27 weeks and maintained on CDAHF diet. After 27 weeks of treatment, 1-h post-dose, blood samples were collected for estimation of non-fasted serum ALT, AST, TG,TC, adiponectin, TNF-α, alpha-fetoprotein (AFP).

Animals were sacrificed and liver was observed for the presence of tumors. Liver was quickly resected, weighed and fixed in 10% formalin for histological analysis or snap-frozen with or without RNAlater solution in liquid nitrogen for RNA sequencing and other biochemical assays like liver lipids (TG and TC) and liver oxidative stress markers like malondialdehyde (MDA).

### Biochemical and histological assessment

Various biochemical markers such as serum ALT, AST, TG, TC, adiponectin, Alphafetoprotein (AFP),osteopontin (OPN),TNF-α,liver lipid and liver MDA levels were estimated using standard procedures mentioned in supplementary information: Annexure I.

### Serum biochemical analysis

Serum ALT,AST,TC and TG levels were determined using commercial kits (Roche Diagnostics, Germany) on a Cobas c311 auto analyzer (Roche, Germany). The serum levels of AFP, adiponectin and TNF-α were measured using a commercial Quantikine ELISA Kit supplied by R&D Systems, Inc., USA and the osteopontin levels were measured using ELISA kits from KINESISDx, Inc., USA.

### Liver biochemistry

Total liver lipids were extracted, and hepatic TG and TC content were quantified using test kits from Agappe Diagnostics, India. Briefly liver tissue was homogenized in thirty volumes of ethanol (Ball Mixer Mill, MM 301, Retsch GmbH, Haan, Germany) for liver lipid (TG and TC) estimations following the method reported by Cool et al. [14]. Samples were vortexed and allowed to settle, and the supernatant was centrifuged at 12,000 rpm for 10 min at room temperature. For the biochemical assays, 10 μl of phosphate-buffered saline (PBS) was added to a flat bottom falcon micro-test 96-well plate followed by 2.5 μl of cleared supernatant. Next, 300 μl of TG reagent or TC reagent (AGAPEE TG/TC kit) was added to the wells, and the plate was incubated at 37 °C for 5 min. The plates were read at 546 nm for TG and 505 nm for TC with a Synergy™ HT Multidetection microplate reader (BioTek Instruments, Inc., Highland Park, Winooski, Vermont 05404–0998 USA). The liver Malondialdehyde (MDA) content levels were measured in tissue homogenates using the QuantiChrom TBARS Assay Kit (BioAssays Systems Inc., USA).

### Gene expression analysis

#### RNA extraction, library construction, and RNA-Seq

Liver tissue samples were collected in RNAlater solution (Sigma-Aldrich Cat# R0901). RNA extraction, library construction and RNA-sequencing were performed as per the procedures described in supplementary information: Annexure-II. Briefly RNA extraction was performed using the RNeasy Mini Kit (QIAGEN, Cat# 74104) and quantified using Qubit RNA Assay HS (Invitrogen, Cat# Q32852). RNA purity and RNA integrity was accessed and the samples were subjected to RNA library preparation using NEB Ultra I RNA-Seq Library Prep kit (NEB, Cat# E7530L). Prepared libraries quantified using Qubit High Sensitivity Assay (Invitrogen, Cat# Q32852). The obtained libraries pooled and diluted to final optimal loading concentration before cluster amplification on the Illumina flow cell. Once the cluster generation was completed, the cluster flow cell was loaded on the Illumina HiSeq 4000 instrument to generate 60 M, 100 bp paired-end reads. The read quality was checked trimmed and the paired-end reads were aligned to the reference mouse genome release downloaded from hisat2 website (GRCm38). The aligned reads are used for estimating the expression of the genes. Differential expression analysis of the raw read counts was performed using DESeq2 (1.16.1). The log2 (foldchange) values were found to be normally distributed. Those genes which were found to have log2(fold change) ≤ − 1 or log2(fold change) ≥ 1 were considered as differentially expressed and those genes which had padj < 0.05 were considered as statistically significant.

### Gene ontology (GO) annotation and pathway analysis

Gene Ontology and Reactome Pathway Annotation were done using Amigo2 Gene Ontology.

### Quantitative real-time polymerase chain reaction [qPCR]

Tumor containing liver samples around 100 mg (3 from each group) were homogenized in RNA-Xpress reagent (HIMEDIA) with polytron homoginizer. Total RNA was extracted from tissue in accordance with the supplier’s instructions. 1 μg total RNA from each sample was taken for first strand cDNA synthesis using Verso cDNA synthesis kit (Thermo scientific). An equal amount of cDNA from each sample was taken for quantitative real-time PCR using ABIprism-7300 (Applied Biosystems). Differentially expressed genes in microarray were selected (Igf2-F 5′-GTACTTCCGGACGACTTCCC − 3′, Igf2-R 5′- CTTTGAGCTCTTTGGCAAGCA – 3′, Cdc20-F 5′- GATCCTTGATGCCCCCGAAA − 3′,Cdc20-R 5′-TGCAGGATGTCACCAGAACC − 3′, Elovl3-F 5′- AATTCTAGGCCTGGTAGGCG − 3′,Elovl3-R 5′-GCAGCGATCTCTTCTGCAGTT-3′, Acot1-F 5′- TTCAAGGGCTGGGAATGGAG − 3′,Acot1-R 5′-TTTCTCGCAGCTGGATTGAAC − 3′, Acot3-F 5′- TGCCCTTGCTTTTGTAACACG − 3′,Acot3-R 5′-GGGAGTTGGTGTTTTCCAGC-3′, Slc10a1-F 5′- TTACCTACAAGGCTGCTGCAA − 3′, Slc10a1-R 5′- AAGGCCAGGTTGTGTAGGAG − 3′, Fabp1-F 5′- GTGGTCCGCAATGAGTTCAC − 3′, Fabp1-R 5′- CACCTTCCAGCTTGACGACT − 3′, Col5A2-F 5′- TGGAGCAGTTGGCCCATTAG − 3′, Col5A2-R 5′- CCCAGGCAGTCCAGTTATCC -3′, ADAM8-F 5′- AACAAGCAGCGTCTACGAGC -3′, ADAM8-R 5′- TCTCGGAGCCTTTCGGTAGA-3′, Timp1- F 5′- GTGCACAGTGTTTCCCTGTT − 3′, Timp1- R 5′- AGTGACGGCTCTGGTAGTC − 3′, Col1a1-F 5′- TGATGGGGAAGCTGGCAAG − 3′, Col1a1-R 5′- GAAGCCTCGGTGTCCCTTC − 3′, Tgfb1-F 5′- ATTGCTGTCCCGTGCAGAG − 3′, Tgfb1-R 5′- TCAGCAGCCGGTTACCAAG -3′, MMP13 –F 5′- ACGAGCATCCATCCCGAGACCT -3′,

MMP13 –R 5′- GTGAACCGCAGCACTGAGCCT − 3′) and quantified using Kapa SYBR FAST (KAPA, USA, KK4618). Ribosomal acidic protein (F:5′- TACAGCTTCACCACCACAGC − 3′ and R:5′- TCTCCAGGGAGGAAGAGGAT − 3′) was used as an internal control for normalization of the results and fold change was calculated with ∆∆Ct method.

### Necropsy examination and histological assessment

At the end of the experiment, the mice were sacrificed with asphyxiation using overdose of isoflurane, and a detailed gross pathological examination was performed. The liver was observed grossly for lesions and the number of tumors was counted. The liver and spleen were dissected, fat was removed and the organ weights were recorded. One portion of liver was collected in RNAlater solution for gene expression and another portion was collected and preserved in 10% neutral buffered formalin for histopathological processing. The formalin-fixed liver (all lobes) were trimmed in a way to cover all the grossly observed tumors, processed and paraffin-embedded. Sagittal sections were taken at 3–4 μm. Light microscopic examination of liver tissue was performed using standard hematoxylin and eosin (H&E) staining. In case there were no abnormalities observed in the liver, it was trimmed by following the standard practice as the guidelines [15]. Hepatic fibrosis was assessed using Sirius Red stain. Specimens were scored using the method described by Kleiner et al. [16]. The staging of hepatic fibrosis was classified as stage 0 to 4 (stage 0: none; stage 1: mild perisinusoidal or periportal; stage 2: moderate perisinusoidal or periportal; stage 3: bridging fibrosis; stage 4: cirrhosis). The diagnosis, classification/histological typing, and nomenclature of foci of cellular alteration (FCA), benign and malignant hepatocellular tumors observed in this study were performed as per International Harmonization of Nomenclature and Diagnostic criteria for Lesions in Rats and Mice [17].

### Statistical analysis

For in-vivo studies, data were analysed using one-way analysis of variance (ANOVA), followed by Dunnett’s multiple comparisons test to evaluate the statistical difference between the various treatment groups. *P* < 0.05 was considered significant. # indicates significant difference in control diet (normal control) group versus disease control, *p < 0.05; **p < 0.01, ***p < 0.001 indicates significant difference versus disease control in test compound. All data presented as mean ± SEM. All data analysis was performed using GraphPad Prism software (GraphPad La Jolla California USA).

## Results

### Effect on mortality rate and body weight

We did not find any mortality during the study period. The body weight of mice was maintained in the disease control group while a gradual increase in body weight from base line was observed in normal control group. Saroglitazar at 3 mg/kg dose showed a significant reduction in body weight as compared to disease control group (Table 1).

**Table 1:** Effect on Serum Biochemistry and Hepatic Lipids

Parameters	Normal control	Disease control	D-Saro (1 mg/kg,p.o.)	D-Saro (3 mg/kg,p.o.)
Serum				
ALT (U/L)	54.8 ± 8.1^# #^	350.1 ± 35.4	232.3 ± 36.9*	210.5 ± 31.4**
AST (U/L)	74.8 ± 6.0^# #^	362.2 ± 36.7	231.1 ± 29.1*	203.0 ± 23.8**
TG (mg/dl)	74.0 ± 4.9	69.4 ± 5.2	42.7 ± 3.1***	31.5 ± 3.6***
TC (mg/dl)	194.5 ± 6.3	84.1 ± 5.5	98.1 ± 5.7	78.5 ± 4.2
AFP level (ng/ml)	44.2 ± 3.1^# #^	768.1 ± 136.0	532.8 ± 95.3	447.3 ± 83.5
OPN level (ng/ml)	50.9 ± 3.9	50.5 ± 3.8	65.1 ± 2.6**	65.9 ± 2.9**
Adiponectin (μg/ml)	8.0 ± 0.4^#^	6.0 ± 0.1	9.2 ± 0.9***	14.0 ± 0.2***
TNF-alpha level (pg/ml)	1.1 ± 0.3^# #^	15.0 ± 1.0	5.8 ± 0.5***	4.9 ± 0.6***
Liver				
TG (mg/g of tissue)	50.8 ± 2.2^# #^	102.6 ± 13.7	66.4 ± 8.7*	45.7 ± 8.2***
TC (mg/g of tissue)	4.06 ± 0.5^# #^	20.0 ± 4.2	9.8 ± 2.4*	6.1 ± 1.2**
MDA (μg/gm of tissue)	20.0 ± 1.2^# #^	333.9 ± 59.2	18.1 ± 0.8***	21.9 ± 1.7***

Values are expressed as the means ± SEM (*n* = 6–15). One way ANOVA followed by Dunnett’s multiple comparisons test was applied for statistics. * indicates significance at *p* < 0.05; **p < 0.01, *** *p* < 0.001 versus Disease control group. ^#^ indicates significance of control diet (Normal control) versus Disease control, at *p* < 0.05 and ^# #^ *p* < 0.01. AST- aspartate aminotransferase. *ALT* Alanine aminotransferase, *TG* Triglyceride, *TC* Total cholesterol, *AFP* Alpha fetoprotein, *OPN* Osteopontin, *TNF-alpha* Tumor necrosis factor-alpha, *MDA* Malondialdehyde.

### Effect of saroglitazar on serum biomarkers of NASH

Saroglitazar at 3 mg/kg dose showed a significant reduction in the liver injury markers serum ALT (40%), AST (44%) and TNF-α (67%) levels compared with the disease control group (Table 1). Saroglitazar treatment also showed a significant dose-dependent reduction in serum triglycerides levels (38 and 55% at 1 and 3 mg/kg dose, respectively) (Table 1). The disease control group showed two and five-fold increase in liver triglycerides and cholesterol levels, respectively which was completely reversed by saroglitazar (Table 1).

### Effect of saroglitazar serum and liver biomarkers of HCC

Serum Alpha-fetoprotein (AFP), a well-established HCC biomarker levels were significantly (17- fold) higher in disease control animals as compared to normal control animals (768 ng/mL Vs 44 ng/ml). Saroglitazar treatment showed dose-dependent 31 and 42% reduction in serum AFP levels at 1 and 3 mg/kg dose, respectively (Table 1). Osteopontin (OPN) deficiency is known to enhance the susceptibility to DEN induced tumorigenesis and OPN acts as a protector during inflammatory liver injury. Saroglitazar treatment causes a significant increase in serum osteopontin levels (50 ng/ml to 65 ng/ml) (Table 1). The status of reactive oxygen species (ROS) measured by malondialdehyde (MDA) levels, liver MDA levels were significantly (17-fold) higher in disease control animals as compared to normal control animals, but these high levels of ROS were completely normalized (> 93% reduced) by saroglitazar treatment (Table 1). Decreased serum adiponectin levels are involved in obesity- and diabetes-related liver tumorigenesis [18]. Disease control animals (animals with the tumor) showed a significant decrease in adiponectin levels whereas saroglitazar treatment showed significant (132%) increase in adiponectin levels (Table 1).

### Effects of saroglitazar on DEN-induced liver tumorigenesis

**Fig. 1:**
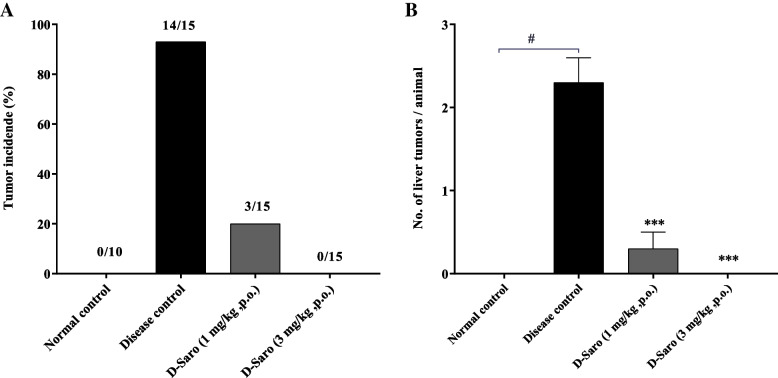
Effect of saroglitazar on tumor incidence and no. of tumors observed after gross examination of liver. Liver of each animals were grossly observed during necropsy for **A**. presence of tumors and **B**. number of tumors per animals were calculated. Values are expressed as the means ± SEM (*n* = 15). #*p* < 0.0001 vs. Normal Control group, ***p < 0.001 vs. Disease Control Vehicle treated group using one way ANOVA followed by Dunnett’s multiple comparison test

**Fig. 2:**
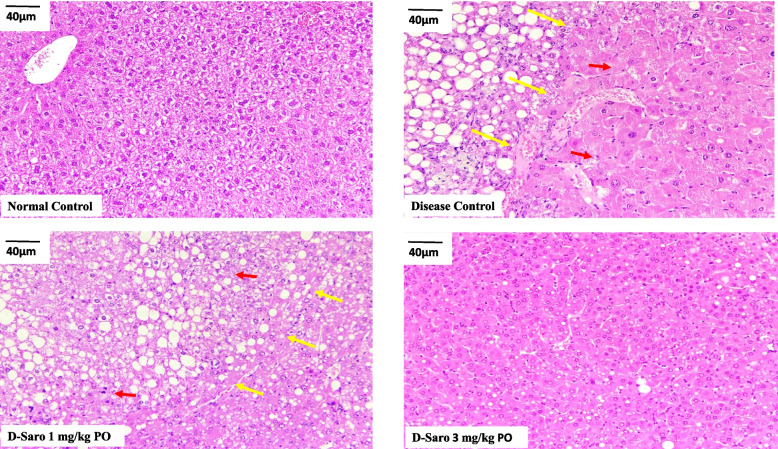
Effect of saroglitazar on liver histology (H&E staining). Representative images of liver sections stained with hematoxylin-eosin and observed at 20X resolution and scale bar 40 μm. Liver sections of animals fed a normal control diet appeared to be normal. Hepatocellular adenoma (Red arrow) and zone of compression (yellow arrow) were observed in disease control, which was completely reversed in animals treated with 3 mg/kg saroglitazar

Macroscopically, whitish and nodular tumors were observed only in the livers of mice from the disease control group. Almost all 14 of 15 (93%) animals in the disease control group showed liver tumors (Fig. 1A) and each liver has 2–3 tumors. The effect of saroglitazar treatment on tumorigenesis seems to be dose dependent, which is 80% reduction in tumor incidence at 1 mg/kg dose and 100% at 3 mg/kg dose (Fig. 1A & 1B). Liver specimens were evaluated using hematoxylin-eosin staining. Histologically hepatic neoplasms including liver cell adenoma and HCC were developed only in disease control mice. Hepatocellular adenomas were found in 9 of 11 (82%) animals in the disease control group, whereas in saroglitazar (3 mg/kg) treatment group none of the animals had hepatocellular adenoma (Fig. 2). There was one animal with hepatocellular carcinoma in the disease control group. Saroglitazar treatment completely inhibited the occurrence of adenoma and HCC (Additional file-1: Table S2).

### Effects of saroglitazar on DEN-induced hepatic fibrosis

**Fig. 3:**
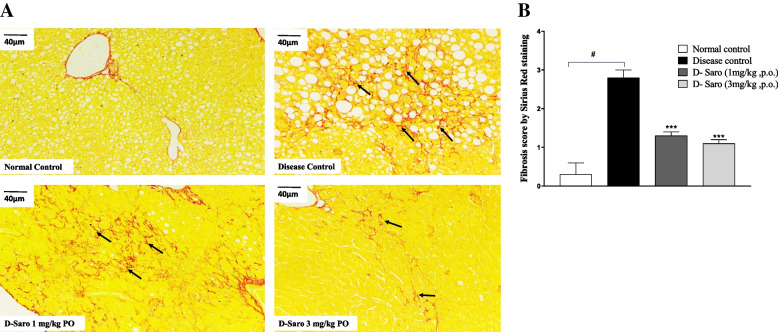
Effect of saroglitazar on liver histology (Sirius Red staining). **A**. Representative images of liver sections stained with Sirius Red staining and observed at 20X resolution and scale bar 40 μm. Liver sections of animals fed a normal control diet appeared to be normal. **B**. A score of 2–3 was assigned to the liver section of disease control animal and was considered to be moderate grade liver fibrosis (black arrow), which was reduced to minimal grade in animals treated with 3 mg/kg saroglitazar (Fig. 3A). # *p* < 0.0001 vs normal control, *** *p* < 0.001 vs disease control

Sirius red stained liver specimens showed moderate grade liver fibrosis in disease control animals which was significantly prevented by saroglitazar treatment at both 1 mg and 3 mg/kg doses (Fig. 3A and B and Additional file-1: Table S2).

### Effects of saroglitazar on DEN-induced liver preneoplastic lesions

In mouse hepatocarcinogenesis, foci of cellular alteration (FCA) have been used as markers of preneoplastic lesions and in short- or medium-term bioassay systems, preneoplastic lesions are evaluated as endpoint markers for hepatocarcinogenicity. Foci of cellular alteration (FCA) represent small to large aggregates of tinctorially distinct hepatocytes within the hepatic parenchyma and are considered preneoplastic lesions. They are classified based upon their phenotypic appearance as basophilic, eosinophilic, clear cell, vacuolated, and mixed foci. The liver preneoplastic lesion was observed in the mice from all groups except normal control at the termination of the experiment. Treatment with a saroglitazar (3 mg/kg) showed a trend to inhibit the development of FCA in comparison to disease control animals (supplementaty information : Table S2).

### Effect of Saroglitazar treatment on the transcriptomic profile using next-generation-sequencing (NGS)

**Fig. 4:**
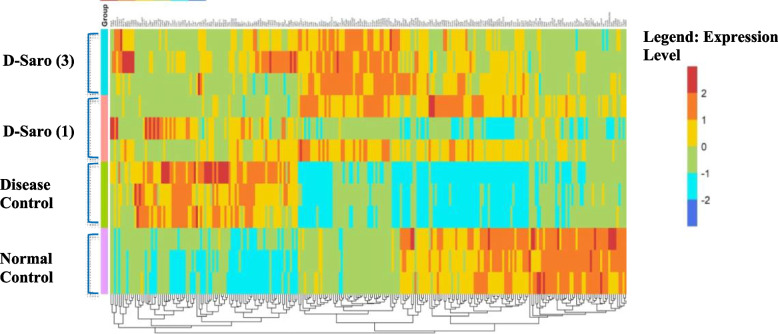
Effect of 27 weeks treatment with saroglitazar on hepatic mRNA levels. Heatmap of liver transcriptomic profile using next-generation sequencing (NGS). Genes that had a *p*-value adjusted (padj) value < 0.05 were considered as significant results in the differential expression analysis. Those genes which were found to have log2 (fold-change) ≤ − 1 or log2(fold-change) ≥ 1 among the significant results, were considered to be significantly down-regulated or up-regulated respectively

Heat map visualization of the hepatic transcriptome demonstrated distinct differences and well segregation between normal control Vs disease control. Saroglitazar treated group pattern had close resemblance towards the normal control group (Fig. 4). The differential expression analysis was performed using DESeq2. Samples were considered as control and test for the differential expression analysis based on the comparisons. The log2 (fold-change) was calculated for the genes based on the raw counts using DESeq2. Genes that had a adjusted *p*-value < 0.05 were considered as significant results in the differential expression analysis. Those genes which were found to have log2(fold-change) ≤ − 1 or log2(fold-change) ≥ 1 among the significant results, were considered to be significantly down-regulated or up-regulated respectively (Fig. 4). In the disease control group total of 2035 genes were significantly upregulated and 1115 genes were significantly downregulated as compared to the normal control group. Saroglitazar treatment (3 mg/kg) caused significant upregulation of 1207 genes while 1319 genes were significantly downregulated as compared to the disease control group (supplemnetaty information: Table S3).

**Fig. 5:**
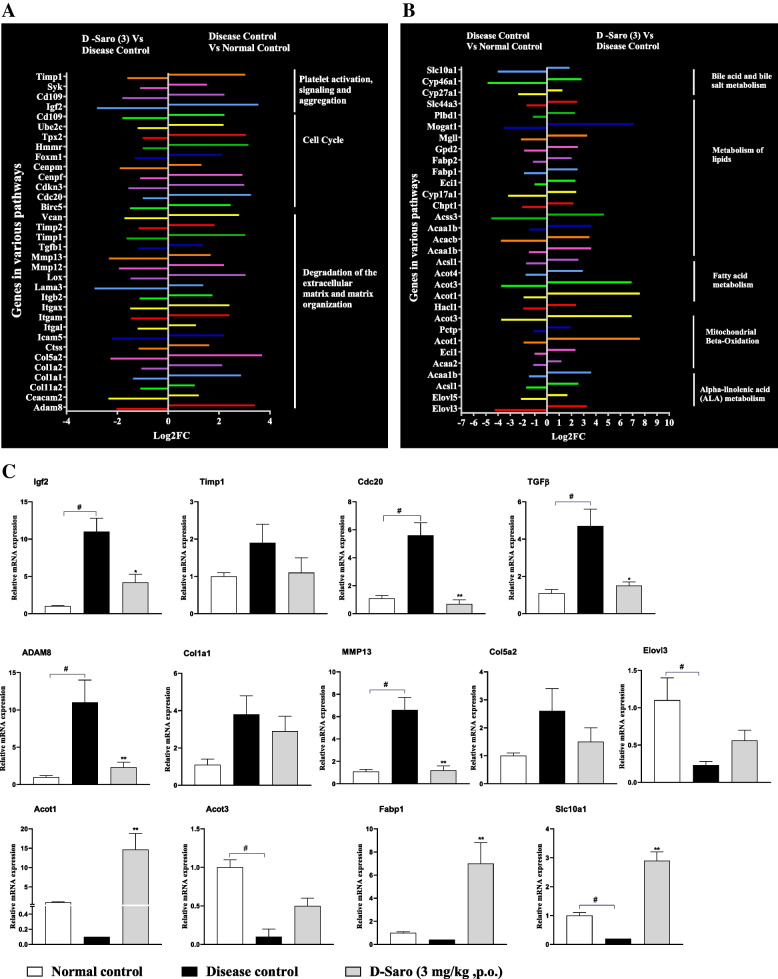
Effect of saroglitazar on different pathways AmiGO2 Gene Ontology was used to perform the GO enrichment and pathway analysis for the given, up or down- regulated genes. **A**. Saroglitazar treatment significantly down regulated the various genes involved in matrix degradation and extracellular matrix organization pathway, cell cycle pathway and platelet activation pathways, which were up regulated in disease control group animals. **B**. Saroglitazar treatment for 27 weeks also significantly up-regulated genes involved in mitochondrial fatty acid beta-oxidation, lipid metabolism, Bile acid/bile salt metabolism and alpha-linolenic acid (ALA) metabolism pathways. **C**. RTPCR data for relative mRNA expression of Igf2, Timp1, Cdc20, TGFβ, ADAM8, Col1a1, Col5a2, Elovl3, Acot1, Acot3, Fabp1, Slc10a1. Values are expressed as the means ± SEM (*n* = 3), * *p* < 0.05,**p < 0.01 vs. Disease Control Vehicle treated group using one way ANOVA followed by Dunnett’s multiple comparison test

AmiGO2 Gene Ontology was used to perform the GO enrichment and Pathway analysis (Table 2) for the given, up or down-regulated genes. Saroglitazar treatment significantly down-regulated the various genes involved in matrix degradation and extracellular matrix organization pathway (*Adam8, Ceacam2, Col11a2, Col1a1, Col1a2, Col5a2, Ctss, Icam5, Itgal, Itgam, Itgax, Itgb2, Lama3, Lox, Mmp12, Mmp13, Tgfb1, Timp1, Timp2*), cell cycle pathway (*Vcan, Birc5, Cdc20, Cdkn3, Cenpf, Cenpm, Foxm1, Hmmr, Tpx2, Ube2c, Cd109*) and platelet activation pathways (*Igf2,Cd109, Syk, Timp1*) which were up-regulated in disease control group animals (Fig. 5A).

**Table 2:** Biochemical pathways altered by Saroglitazar treatment in disease model

Reactome pathways	No. of entities found	Total no. of entities	Fold enrichment	*P*-value	Regulation
Triglyceride catabolism	5	20	19.45	3.1 X 10^−2^	Up
Beta-oxidation of fatty acids	10	39	16.05	4.1 X 10^−5^	Up
Alpha-linolenic acid (ALA) metabolism	9	46	13.41	6.1 X 10^−4^	Up
Mitochondrial Fatty Acid Beta-Oxidation	26	110	11.23	1.4 X 10^−10^	Up
Peroxisomal lipid metabolism)	20	96	10.1	1.5 X 10^−6^	Up
Triglyceride metabolism	10	49	8.69	2.9 X 10^−3^	Up
Peroxisomal protein import	24	80	7.36	3.5 X 10^−9^	Up
Bile acid and bile salt metabolism	9	139	7.32	4.8 X 10^−2^	Up
Fatty acyl-CoA biosynthesis	11	76	7.15	1.5 X 10^−3^	Up
Fatty acid metabolism	83	458	7.02	5.7 X 10^− 28^	Up
Glycerophospholipid biosynthesis	25	247	4.83	2.6 X 10^−7^	Up
Metabolism of lipids	150	1383	4.47	3.1 X 10^−41^	Up
Biological oxidations	60	662	4.12	2.2 X 10^−12^	Up
Metabolism of steroids	20	299	3.69	7.8 X 10^−4^	Up
Phospholipid metabolism	27	378	3.28	1.6 X 10^−4^	Up
Metabolism	274	3564	2.96	1.1 X 10^− 49^	Up
Degradation of the extracellular matrix	10	169	5.85	2.3 X 10^−2^	Down
Extracellular matrix organization	52	393	2.63	3.2 X 10^−3^	Down

Enrichment pathway analysis showed saroglitazar treatment significantly up-regulated genes involved in mitochondrial fatty acid beta-oxidation (*Acaa2,Eci1,Acot1,Pctp,Acot3, Hacl1, Acot1, Acot4,Acsl1*), lipid metabolism (*Acaa1b, Acss3, Chpt1, Cyp17a1, Eci1, Fabp1, Fabp2, Gpd2, Mgll, Mogat1, Plbd1*), Bile acid/bile salt metabolism (*Slc44a3, Cyp27a1, Cyp46a1, Slc10a1*) and alpha-linolenic acid (ALA) metabolism (*Elovl3, Elovl5, Acsl1, Acaa1b*) pathways (Fig. 5B), which are involved in prevention of HCC through induction of apoptosis, antioxidant activity, limiting the cell proliferation and inhibition of growth signal transduction. When we analysed the cancer-specific genes like *Cdc20, Hmmr, Tpx2, Cenpf, Birc5, Ube2c, Foxm1,Pold4, Nup210, Cenpm and Orc1*, these pro-carcinogenic genes found to be over expressed in the disease control group. Saroglitazar treatment showed down-regulation in the expression of above mentioned cancer related genes as represented in Table 3.

**Table 3:** Effect on hepatic mRNA expresion of cancer related genes using RNA-seq analysis

Sr.no	Gene symbol	Normal control Vs Disease control log2 Fold Change	Disease control Vs D-Saro (3 mg/kg) log2 Fold Change	Reference link for significance of genes in HCC
1	**Cdc20**	3.25	-1	https://www.spandidos-publications.com/10.3892/mmr.2021.12122
2	**Hmmr**	3.15	-1	https://www.spandidos-publications.com/10.3892/ol.2020.11844#:~:text=Hyaluronan%20mediated%20motility%20receptor%20
3	**Tpx2**	3.05	-1	https://www.ncbi.nlm.nih.gov/pmc/articles/PMC6844623/
4	**Cenpf**	2.91	−1.1	https://www.frontiersin.org/articles/10.3389/fonc.2021.738841/full
5	**Birc5**	2.45	−1.5	https://bmccancer.biomedcentral.com/articles/10.1186/s12885-021-08390-7
6	**Ube2c**	2.17	−1.2	https://www.ncbi.nlm.nih.gov/pmc/articles/PMC6470407/
7	**Foxm1**	2.1	−1.3	https://www.nature.com/articles/emm2017159
8	**Pold4**	1.46	−1.1	https://www.nature.com/articles/s41467-018-06931-6
9	**Nup210**	1.39	−1.1	https://aacrjournals.org/cancerres/article/81/2/356/648678/Nucleoporin-210-Serves-a-Key-Scaffold-for-SMARCB1
10	**Prkcb**	1.38	−1.2	https://pubmed.ncbi.nlm.nih.gov/18167130/
11	**Cenpm**	1.3	−1.9	https://jeccr.biomedcentral.com/articles/10.1186/s13046-019-1444-0
12	**Orc1**	1.25	−1.1	https://www.ncbi.nlm.nih.gov/pmc/articles/PMC7052853/

### Effect of Saroglitazar treatment on gene expression profile using qPCR

Gene expression analysis using RNA-Sequencing showed that disease control animals showed upregualtion of various genes involved in matrix degradation and extracellular matrix organization pathway (*Adam8, Col1a1, Col5a2, Mmp13, Tgfb1, Timp1, Timp2*), cell cycle pathway (*Cdc20,)* and platelet activation pathways (*Igf2, Timp1*) and those genes were down-regualted in Saroglitazar treatment. It also showed sarogliazar treatment causes up-regulation of genes involved in mitochondrial fatty acid beta-oxidation (*Acot1, Acot3,),* lipid metabolism (*Fabp1*), Bile acid/bile salt metabolism (*Slc10a1*) and alpha-linolenic acid (ALA) metabolism (*Elovl3,)* pathways (Fig. 5B), which are involved in prevention of HCC through induction of apoptosis, antioxidant activity, limiting the cell proliferation and inhibition of growth signal transduction. All these gene expression was further confirmed by qPCR analysis and fold change Vs nomral control is given in Fig. 5C. The qPCR data has confirmed the oberservations of RNA-seq analysis. Saroglitazar treatment significantly down-regulated the various genes involved in matrix degradation and extracellular matrix organization pathway and platelet activation pathways which were up-regulated in disease control group animals (Fig. 5C) and Saroglitazar causes up-regulation of genes involved in mitochondrial fatty acid beta-oxidation and, lipid metabolism pathways (Fig. 5C).

## Discussion

Due to its high prevalence and progressive form, NAFLD has become a leading etiology for hepatocellular carcinoma (HCC). Hepatic lipid accumulation leads to metabolic reprogramming, characterized by a combination of cellular metabolic alterations and an accumulation of potentially toxic metabolites that favor the development of liver tumorigenesis [19] .

Saroglitazar is known to affect multiple metabolic pathways, thereby improving insulin sensitivity and reducing serum and liver lipids [7, 8, 20]. Additionally, saroglitazar showed several pleiotropic effects. It showed improvement of NAFLD/NASH [7, 10, 21]. In line with these observations, we report here that saroglitazar could prevent NASH development and the progression to liver tumorigenesis. HCC initiated by DEN injection in mice is widely used and well-characterized model as it recapitulates aspects of liver injury, fibrosis and hepatitis which forms the basis of human HCC [22] and is comparable to its human counterpart in terms of cancer-associated gene expression patterns and carcinogenetic pathways. It is considered among the best-fit experimental models of HCC [23]. The choline-deficient, L-amino acid-defined, high-fat diet (CDAHFD) model mimics human NASH by sequentially producing steatohepatitis, liver fibrosis, and liver cancer [7, 24] and combining the two approaches, i.e., feeding C57BL/6 mice a choline-deficient, L-amino acid-defined, high-fat diet (CDAHFD) with DEN exposure a novel experimental NASH-HCC mouse model that exhibited key clinical features, including inflammation, fibrosis, and carcinogenesis was developed and used for this study.

Saroglitazar is a PPAR-α/γ dual agonist, having predominant PPAR-α activity [25]. PPARs act as intracellular lipid sensors that coordinate genetic networks regulating lipid metabolism, energy utilization and they also have roles in mechanisms of cell cycling, anti-inflammatory responses and apoptosis [26]. The role of PPARs in liver cancer is controversial [11, 27]; it is not clear whether PPAR agonists would promote cancer or control it. Several preclinical studies are suggesting that PPAR activation can potentiate tumorigenesis [11]. In contrast, several in-vitro models have reported antiproliferation properties for PPAR agonists [28]. PPAR-α agonists (fibrates) are known to induce liver-specific peroxisomal proliferation, hepatomegaly, and ultimately hepatocellular carcinoma in rodents; however, this response is not observed in higher species. In non-human primates, PPAR-α agonists neither induced peroxisome proliferation nor liver tumor development [13, 29, 30]. These observations are supported by the absence of association between liver cancer and PPAR alpha agonist (fibrates) in humans for several decades [11].

As far as rodents are concerned, we are reporting for the first time that a potent PPAR-α agonist like saroglitazar could inhibit liver tumors instead of causing them as expected from PPAR agonists in rodents. In this study, chronic treatment with saroglitazar completely prevented the induction of liver tumors by DEN & CDAHFD administration. Our data indicate that rodent‘s response having NAFLD-NASH to saroglitazar treatment is different from that of normal rodents. While in normal rats, PPAR agonists cause peroxisome proliferation, thereby enhancing the potential for liver tumor formation, on the other hand, in NAFLD-NASH models, the tumor is induced by a combination of factors that appears to be blocked by saroglitazar in this study. In normal Wistar rats, saroglitazar treatment showed elevated serum alanine aminotransferase (ALT) levels and higher liver weights with histologically hepatocellular hypertrophy and minimal to mild single cell necrosis in the liver, which are classical rodent-specific PPAR-αmediated peroxisome proliferation-related effects [25]. Whereas in various NASH animal models, saroglitazar treatment showed a significant reduction in ALT and histologically improved steatosis, inflammation, ballooning and fibrosis in the liver [6, 7]. Consistent with the above findings, in this work, we have found that saroglitazar treatment caused a significant reduction in serum ALT and AST and histological improvement in NASH and the prevention of tumors.

The tumor-suppressive effect of saroglitazar on the development of NASH-induced liver tumorigenesis was most likely associated with the alteration in metabolic programming due to PPAR activation leading to improvement in hepatic steatosis (Fig. 2), attenuation of inflammation, and oxidative stress (Table 1). It is known that insulin resistance leads to excess accumulation of lipids in the liver. Lipotoxicity, coupled with oxidative stress and inflammation, induces & accelerates hepatic tumorigenesis [31]. Saroglitazar improves insulin sensitivity and ameliorates hepatic steatosis by decreasing fatty acid influx to the liver and enhancing fatty acid oxidation [7]. In this regard, this study highlights the anti-inflammatory, antioxidant, and anti-fibrotic activity of saroglitazar. The transcriptomic analysis further supported this. Saroglitazar treatment significantly down-regulated the matrix degradation and extracellular matrix organization, cell cycle, and platelet activation pathways, which were up-regulated in disease control group animals and were responsible for increased cell proliferation, growth, and tumor invasion. Whereas, saroglitazar treatment significantly up-regulated alpha-linolenic acid (ALA) metabolism, mitochondrial beta-oxidation, and lipid metabolism pathways, which are involved in preventing HCC through induction of apoptosis, antioxidant activity, limiting the cell proliferation and inhibition of growth signal transduction (Table 2). Overall the genes involved in tumor progression and invasion were downregulated, whereas; genes involved in apoptosis, antioxidant activity and limiting the cell proliferation were up-regulated by saroglitazar treatment.

Reactive oxygen species (ROS) accumulation in livers results in depletion of cellular antioxidants and increased lipid peroxidation in NASH controls. Saroglitazar treatment completely normalized the high levels of ROS as measured by MDA levels. Decreased adiponectin levels are critically involved in obesity- and diabetes-related liver tumorigenesis [18, 32] Saroglitazar treatment has increased the adiponectin levels, so this may be one mechanism for preventing liver tumorigenesis. Osteopontin (OPN) deficiency enhanced the susceptibility to DEN-induced tumorigenesis by promoting the liver injury via augmenting oxidative stress [33, 34]. OPN acts as a protector during inflammatory liver injury [34]. Saroglitazar treatment causes a significant increase in serum osteopontin levels.

It is well known that many factors limit the translation of preclinical data to humans. The model chosen for this study has been validated to recapitulate the key elements of human disease of NASH-related HCC [22, 23]. But in human, although a large proportion of the population (24%) has NAFLD, only a minority (incidence of 2.4–12.8%) will exhibit progressive liver disease or experience a liver-related death [35] and secondly human NASH is extremely heterogeneous [36] unlike in mice most animals treated DEN produced liver tumors, so these factors need to be taken care of while correlating this study’s findings to humans.

## Conclusion

In conclusion, in this study, we have shown that saroglitazar effectively prevents NASH’s development and progression to liver tumorigenesis by inhibiting steatosis, fibrosis, and inflammatory pathways while improving adipokine imbalance and mitochondrial dysfunction. These data suggest that saroglitazar may help to prevent HCC in patients with NAFLD/NASH by reprograming the metabolic pathways.

## Supplementary Information

**Additional file 1: Annexure I.** Method of serum and liver biochemical analysis. **Annexure II.** Method of gene expression analysis. **Table S1.** Serum ALT and AST levels after 8 week CDAHFD feeding along with a single intraperitoneal dose of DEN at 4 week of age. **Table S2.** Liver Histology Scores and Tumor Incidence. **Table S3.** List of differentially expressed genes. **Table S4.** Dietary composition of Diet used for creating NASH in C57 mice. **Fig. F1.** Structural formula of Saroglitazar. **Annexure III.** Raw data for serum and liver biomarkers and liver histology.

## Data Availability

All data generated or analyzed during this study are included in this submitted manuscript, [raw data and other supporting information are included in its supplementary material as additional file in Annexure III].

## Abbreviations

CDAHFD: Cholinedeficient, l-amino acid- defined, high-fat diet
HCC: Hepatocellular carcinoma
NAFLD: Nonalcoholic fatty liver disease
NASH: Nonalcoholic steatohepatitis
ALT: Alanine aminotransferase
AST: Aspartate aminotransferase
TG: Triglyceride
TC: Total cholesterol
TNF-α: Tumor necrosis factor
AFP: Alpha-fetoprotein
MDA: Malondialdehyde
