# Supplementary material for: Saroglitazar suppresses the hepatocellular carcinoma induced by intraperitoneal injection of diethylnitrosamine in C57BL/6 mice fed on choline deficient, l-amino acid- defined, high-fat diet
Source: BMC Cancer. 2023 Jan 17;23:59. doi: 10.1186/s12885-023-10530-0 (PMC9843913; doi:10.1186/s12885-023-10530-0)
Supplement: Supplementary file 1 — Additional file 1: Annexure I. Method of serum and liver biochemical analysis. Annexure II. Method of gene expression analysis. Table S1. Serum ALT and AST levels after 8 week CDAHFD feeding along with a single intraperitoneal dose of DEN at 4 week of age. Table S2. Liver Histology Scores and Tumor Incidence. Table S3. List of differentially expressed genes. Table S4. Dietary composition of Diet used for creating NASH in C57 mice. Fig. F1. Structural formula of Saroglitazar. Annexure III. Raw data for serum and liver biomarkers and liver histology. [file 12885_2023_10530_MOESM1_ESM.docx]

**Additional file 1:** **Annexure I**

***Biochemical analysis***

*Serum analysis.*

ALT, AST and triglyceride (TG) levels were determined using commercial kits (Roche Diagnostics, Germany) on a Cobas c311 autoanalyzer (Roche, Germany). Total liver lipids were extracted, and hepatic TG and total cholesterol (TC) content was quantified using triglyceride and total cholesterol test kits from Agappe Diagnostics, India.

*Liver biochemistry.*

Liver tissue was homogenized in thirty volumes of ethanol (Ball Mixer Mill, MM 301, Retsch GmbH, Haan, Germany) for liver lipid (TG and TC) estimations following the method reported by Cool et al. (1). Samples were vortexed and allowed to settle, and the supernatant was centrifuged at 12,000 rpm for 10 min at room temperature. For the biochemical assays, 10 μl of phosphate buffered saline (PBS) added to a flat bottom falcon micro test 96-well plate followed by 2.5 μl of cleared supernatant. Next, 300 μl of TG reagent or TC reagent (AGAPEE TG/TC kit) was added to the wells, and the plate was incubated at 37ºC for 5 min. The plates were read at 546 nm for TG and 505 nm for TC with a Synergy™ HT Multi-detection microplate reader (BioTek Instruments, Inc., Highland Park, Winooski, Vermont 05404-0998 USA)..

The liver Malondialdehyde (MDA) content levels were measured in tissue homogenates using QuantiChrom TBARS Assay Kit (BioAssays Systems Inc, USA).

**Additional file-1:** **Annexure II**

**Materials and Methods**

**Gene expression analysis**

RNA from 28 tissue samples were extracted by using RNeasy Mini Kit (QIAGEN, Cat# 74104) and quantified using Qubit RNA Assay HS (Invitrogen, Cat# Q32852). RNA purity was checked using QIAxpert (Qiagen, Cat# 9002368) and RNA integrity was assessed on TapeStation using RNA HS ScreenTapes (Agilent, Cat# 5067-5579). Of 28 samples, 12 samples were confirmed to proceed for the RNA library prep.

**RNA Library Prep Protocol**

NEB Ultra I RNA-Seq Library Prep kit protocol (NEB, Cat# E7530L) was used to prepare libraries for total RNA sequencing. First, the ribosomal RNA (rRNA) which constitutes for ~95% of the total RNA population were removed using biotinylated, target-specific oligos combined with Ribo-Zero rRNA removal beads (Lexogen, Cat# 037.96). Following purification, the ribodepleted RNA was fragmented using divalent captions under elevated temperatures. The cleaved RNA fragments were copied into first strand cDNA using reverse transcriptase. Second strand cDNA synthesis was performed, using DNA Polymerase I and RNase H enzyme. The cDNA fragments were then subjected to a series of enzymatic steps which repair the ends, tails the 3’ end with a single ‘A’ base, followed by ligation of the adapters. The adapter ligated products were then purified and enriched using the following thermal conditions: initial denaturation 98°C for 30sec; 12 cycles of - 98°C for 10sec, 65°C for 75sec; final extension of 65°C for 5mins. PCR products are then purified and checked for fragment size distribution on TapeStation using D1000 DNA Screen Tapes (Agilent, Cat# 5067-5582).

Prepared libraries were quantified using Qubit High Sensitivity Assay (Invitrogen, Cat# Q32852). The obtained libraries were pooled and diluted to final optimal loading concentration before cluster amplification on Illumina flow cell. Once the cluster generation is completed, the cluster flow cell is loaded on Illumina HiSeq 4000 instrument to generate 60M, 100bp paired end reads.

**Read quality check**

We check the following parameters from fastq file. Base quality score distribution . Sequence quality score distribution. Average base content per read. GC distribution in the reads. PCR amplification issue . Check for over-represented sequences.

**Adapter trimming**

Based on quality report of fastq files we trim sequence read where necessary to only retain high quality sequence for further analysis. In addition, the low-quality sequence reads are excluded from the analysis. The adapter trimming was performed using Trimmomatic (v-0.36).

**Contamination removal**

For the RNA-Seq analysis we begin by removing the unwanted sequences, especially nonpolyA tailed RNAs from the sample (assuming that poly-A tailed RNAs are sequenced). The unwanted sequences include - mitochondrial genome sequences, ribosomal RNAs, transfer RNAs, adapter sequences and others. Contamination removal was performed using Bowtie2 (2.2.4).

**Read alignment**

The paired-end reads are aligned to the reference mouse genome release downloaded from hisat2 website (GRCm38). The reference genome file was downloaded from the following website (FTP://FTP.CCB.JHU.EDU/PUB/INFPHILO/HISAT2/DATA/GRCM38.TAR.GZ). GTF file was downloaded from the following website (FTP://FTP.ENSEMBL.ORG/PUB/RELEASE78/GTF/MUS_MUSCULUS/MUS_MUSCULUS.GRCM38.78.GTF.GZ). Alignment was performed using HISAT2 (2.1.0). Qualimap 2.2.1 was used to obtain the sequence alignment distribution of the aligned reads. RSeQC (2.3.7) was used to find out the splice junction distribution across the aligned reads.

**Expression estimation**

The aligned reads are used for estimating expression of the genes. The raw read counts were estimated using featureCounts (1.5.2). Cufflinks (2.2.1) was used to calculate the gene expression values for mouse genes and isoforms of the gtf file (fpkm values) using the aligned reads.

**Differential expression**

Differential expression analysis of the raw read counts was performed using DESeq2 (1.16.1). A distribution of these log2 (fold-change) values were found to be normally distributed. Those genes which were found to have log2(fold change) ≤ -1 or log2(fold change) ≥ 1 were considered as differentially expressed and those genes which had padj < 0.05 were considered as statistically significant.

**Additional file-1:** Table S1- Serum ALT and AST levels after 8 week CDAHFD feeding along with a single intraperitoneal dose of DEN at 4 week of age (n=10/15).

| **Treatment** | **Serum ALT(U/L)** | **Serum AST (U/L)** |
| --- | --- | --- |
| Normal Control | 36.5 ± 10.1 | 63.6 ± 11.2 |
| Disease control | 359.9 ± 14.5 | 282.4 ± 10.7 |
| D-Saro (1 mg/kg,p.o.) | 362.0 ± 15.2 | 290.5 ± 11.6 |
| D-Saro (3 mg/kg,p.o.) | 362.6 ± 15.2 | 289.2 ± 11.9 |

**Additional file-1:** Table S2- Liver Histology Scores and Tumor Incidence

| **Treatment** | **Fibrosis Score (Sirius Red staining)** | **No. of eosinophilic foci** | **No. of basophilic foci** | | **No. of clear Cell foci** | **No. of mixed cell foci** | **No. of hepatocellular adenoma** | **Tumor incidence (%)** | **No. of tumors per animal** |
| --- | --- | --- | --- | --- | --- | --- | --- | --- | --- |
| Normal Control | 0.3 ± 0.3 | 0.0 ± 0.0 | 0.0 ± 0.0 | 0.3 ± 0.3 | | 0.3 ± 0.3 | 0.2 ± 0.2 | 0 (0 out of 15) | 0.0 ± 0.0 |
| Disease control | 2.8 ± 0.2 | 3.3 ± 0.5 | 2.5 ± 0.9 | 2.5 ± 0.8 | | 2.1 ± 0.7 | 3.1 ± 0.7 | 93.3 (14 out of 15) | 2.3 ± 0.3 |
| D-Saro (1 mg/kg,p.o.) | 1.3 ± 0.1 | 2.9 ± 0.6 | 3.0 ± 0.7 | 1.7 ±0.5 | | 1.2 ± 0.4 | 0.4 ± 0.2 | 20.0 (3 out of 15) | 0.3 ± 0.2 |
| D-Saro (3 mg/kg,p.o.) | 1.1 ± 0.1 | 2.8 ± 0.6 | 3.0 ± 0.8 | 1.0 ± 0.6 | | 1.1 ± 0.3 | 0.0 ± 0.0 | 0.0 (0 out of 15) | 0.0 ± 0.0 |

**Additional file-1:** Table S3- List of differentially expressed genes

| **Comparison** | **Number of Significant Up-regulated genes** | **Number of Significant Down-regulated genes** |
| --- | --- | --- |
| Disease Control Vs Normal Control | 2035.0 | 1115.0 |
| D-Saro (1 mg/kg,p.o.) Vs Disease Control | 518.0 | 299.0 |
| D-Saro (3 mg/kg,p.o.) Vs Disease Control | 1207.0 | 1319.0 |
| D-Saro (3 mg/kg,p.o.) Vs D-Saro (1mg/kg,p.o.) | 96.0 | 62.0 |

**Additional file-1:** Table S4-Dietary composition of Diet used for creating NASH in C57 mice.

| **Product No.** | **A06071309** | | A06071322 | |
| --- | --- | --- | --- | --- |
| **Contents** | gm % | kcal % | gm % | kcal % |
| Protein | 20.8 | 18 | 18.3 | 19.0 |
| Carbohydrate | 42.9 | 36 | 68.0 | 71.0 |
| Fat | 24.1 | 46 | 4.3 | 10.0 |
| **Total** | **85.1** | **100** | **90.7** | **100.0** |
| **kcal/gm** | 5.2 | | 3.8 | |
|  |  |  |  |  |
| **Ingredients** | **gm** | | **gm** | |
| L-Cystine | 4.2 | | 7.0 | |
| L-Isoleucine | 7.6 | | 8.8 | |
| L-Leucine | 15.8 | | 16.2 | |
| L-Lysine | 13.2 | | 13.6 | |
| L- Methionine | 0.8 | | 4.8 | |
| L-Phenylalanine | 8.4 | | 9.2 | |
| L-Threonine | 7.2 | | 7.0 | |
| L-Tryptophan | 2.1 | | 2.2 | |
| L-Valine | 9.3 | | 10.5 | |
| L-Histidine | 4.6 | | 4.8 | |
| L-Alanine | 5.1 | | 4.8 | |
| L-Arginine | 6.0 | | 6.6 | |
| L-Asparatic Acid | 12.1 | | 11.9 | |
| L-Glutamic Acid | 38.2 | | 38.7 | |
| Glycine | 3.0 | | 3.3 | |
| L-Proline | 17.8 | | 21.1 | |
| L-Serine | 10.0 | | 9.9 | |
| L-Tyrosine | 9.2 | | 9.8 | |
| Corn Starch | 0.0 | | 315.0 | |
| Maltodextrin 10 | 130.1 | | 35.0 | |
| Sucrose | 68.8 | | 350.0 | |
| Cellulose, BW200 | 50.0 | | 50.0 | |
| Soybean Oil | 25.0 | | 25.0 | |
| Lard | 245.0 | | 20.0 | |
| Mineral Mix S10026 | 10.0 | | 10.0 | |
| DiCalcium Phosphate | 13.0 | | 13.0 | |
| Calcium Carbonate | 5.5 | | 5.5 | |
| Potassium Citrate | 16.5 | | 16.5 | |
| Sodium Bicarbonate | 7.5 | | - | |
| Vitamin Mix V10001 | 10.0 | | 10.0 | |
| Choline Bitartrate | 0.0 | | 2.0 | |
| **Total** | **755.8** | | **1042.2** | |

**Additional file-1: Figure F1-** Structural formula of Saroglitazar

**Annexure III:**

**Raw data for Table-1 (serum biochemistry) (n=10/15)**

**Annexure III continued..**

**Raw data for Table-1 (serum biomarkers) (n=5/6)**

**Annexure III continued..**

**Raw data for Table-1 (liver biomarkers) (n=5/7)**

**Annexure III continued..**

**Raw data for gross histopathological observation (n=10/15)**

**References:**

1. Cool B, Zinker B, Chiou W, Kifle L, Cao N, Perham M, et al. Identification and characterization of a small molecule AMPK activator that treats key components of type 2 diabetes and the metabolic syndrome. Cell Metab. 2006;3(6):403–16.
